# The Role of MicroRNAs in Regulatory T Cells

**DOI:** 10.1155/2020/3232061

**Published:** 2020-04-02

**Authors:** Chao Liu, Nannan Li, Guijian Liu

**Affiliations:** ^1^Clinical Laboratory, Guang'anmen Hospital, China Academy of Chinese Medical Sciences, China; ^2^Feiyan Biotechnology Company Limited, Beijing, China

## Abstract

MicroRNAs are a class of conserved, 20 nt-23 nt long, noncoding small RNAs that inhibit expression of their respective target genes in different cell types. Regulatory T cells (Tregs) are a subpopulation of T cells that negatively regulate immune responses, which is essential to immune homeostasis. Recent studies have indicated that microRNAs play an important role in the proliferation, differentiation, and functions of Treg. Here, we review the recent progress in understanding the roles of microRNAs in Treg and their dysregulation in immune-related diseases. This ongoing research continues to expand the understanding of Treg regulation and the mechanisms of immune disorders.

## 1. Introduction

MicroRNAs are noncoding, single-stranded, small RNAs with a length of 20-23 nucleotides and were first discovered in *Caenorhabditis elegans* in 1993 [1]. After their discovery, microRNAs gained extensive attention due to their role in regulating gene expression. They were shown to posttranscriptionally downregulate gene expression by specifically binding to the 3′-UTR of target genes. MicroRNAs are common and abundant in both plants and animals [2, 3]. In the miRBase database (the miRBase database, released 19, August 2012), 25,141 mature miRNAs in 193 species have been identified, including 2,042 human mature miRNAs and 1,281 mouse mature miRNAs. There has been evidence demonstrating that microRNAs are involved in many cellular processes such as proliferation, differentiation, and apoptosis [4, 5]. As such, their dysregulation could lead to many diseases, such as cancer, cardiovascular diseases, and diabetes [6–8]. A growing body of recent research has indicated that microRNAs play an important role in controlling the development and function of different immune cell types [9]. The CD4^+^CD25^+^Foxp3^+^ Tregs are a subset of CD4^+^ T lymphocytes and are indispensable for immune homeostasis and self-tolerance. In this review, we summarized the recent progress in understanding the microRNA-mediated regulation in Treg and the role of its dysregulation in disease development.

## 2. MicroRNA Biogenesis

**Figure 1:**
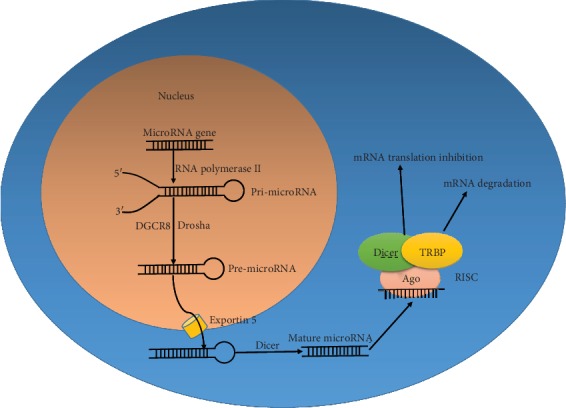
The process of microRNA biogenesis. MicroRNA genes are first transcribed into pri-microRNA by RNA polymerase II. In the nucleus, pri-microRNAs are cleaved by an RNase III enzyme called Drosha and its cofactor DGCR8 into pre-microRNAs. Then, the pre-microRNA is exported into the cytoplasm by Exportin 5. In the cytoplasm, the pre-microRNA is cleaved by another RNase III enzyme Dicer to produce a mature 22 nucleotide long double-stranded microRNA duplex. The functional strand of the microRNA is assembled into RISC, which leads to mRNA degradation or inhibition of translation of its target mRNA.

Most mammalian microRNA genes are located throughout the entire genome and account for approximately 1% of the genome [10]. MicroRNA genes are housed within the protein-coding or noncoding genes and are often located in clusters that may undergo a polycistronic transcription [11, 12]. MicroRNA genes are transcribed by RNA polymerase II into primary transcripts, termed pri-miRNA, and have a 5′ 7-methyl guanosine cap and a 3′ poly-adenylated hairpin structure [13]. In the nucleus, the pri-microRNA is cleaved into a 60-70 nucleotide precursor miRNA (pre-miRNA) by a microprocessor which includes the RNase III enzyme Drosha and its cofactor DGCR8 [14]. Subsequently, pre-miRNAs are exported from the nucleus into the cytoplasm by a dsRNA-binding protein Exportin 5 in a Ran GTP-independent manner [15]. In the cytoplasm, another RNase III enzyme, Dicer, acts on the pre-miRNA to generate mature 22 nucleotide long double-stranded microRNA duplexes [16]. Then, the functional strand of the duplex is assembled into the RNA-induced silencing complex (RISC), which includes Dicer, TRBP, and the Argonaute proteins, while the other strand is released and degraded [17]. The Argonaute proteins provide a suitable circumstance for the microRNA to interact with its target mRNAs. The RISC specifically recognizes mRNAs by base pairing between the position 2-8 nucleotides of microRNAs, known as the seed region, and the complementary 3′-UTR of its target mRNAs [18, 19]. Partial binding between the microRNA and mRNA results in translation inhibition or destruction of the mRNA. An imperfect match between the microRNA and mRNA enables a microRNA to regulate a range of different genes and for a given gene to be regulated by several microRNAs [20] (Figure 1).

## 3. Generation and Immune Suppression Mechanism of the CD4^+^CD25^+^Foxp3^+^ Tregs

Regulatory T cells (Tregs) play a crucial suppressive role during immune responses and are essential for peripheral tolerance and immune homeostasis. Their dysfunction or overabundance could lead to a range of immune-related diseases and cancers. Tregs were first reported in 1995 as a subpopulation of the CD4^+^ T lymphocytes expressing IL-2 receptor *α*-chain (CD25) capable of suppressing responses towards self-antigens [21]. However, they lack a specific marker to differentiate them from other CD4^+^ T cells. In 2003, the transcription factor Foxp3 was found to be highly and specifically expressed in Treg, which enabled Treg to be characterized as the CD4^+^CD25^+^Foxp3^+^ T lymphocytes [22]. Subsequent research indicated that Foxp3 was crucial to the suppressive function of Treg. When Foxp3 was ectopically expressed, it could induce regulatory function in naive T cells [23]. Its deficiency results in failure to generate Treg and a lethal lymphoproliferative disease in mice [24]. In humans, Foxp3 mutations lead to a Treg dysfunction and an immune dysfunction, polyendocrinopathy, enteropathy, and the X-linked (IPEX) syndrome [25]. CD4^+^CD25^+^Foxp3^+^ Tregs can be divided into two classes: natural Tregs (nTregs) and induced Tregs (iTregs). Natural Tregs are generated during T lymphocyte development in the thymus upon high avidity of interaction between TCRs and self-antigens [26]. iTregs arise from the CD4^+^CD25^−^ precursor T cells in the periphery upon antigen presentation by the nonprofessional antigen-presenting cells [27]. Tregs exert their regulatory role through multiple mechanisms including secretion of the inhibitory cytokines, such as interleukin-10, interleukin-35, and transforming growth factor *β*; cytolysis of target cells mediated by granzyme-A, granzyme-B, and perforin; metabolic disruption induced by CD25 and cyclic AMP; and inhibition through modulation of DC maturation by CTLA-4 [28]. Among these, CTLA4- and IL-2-mediated suppression may be the core mechanism [29].

## 4. Research on the Role of MicroRNAs in Tregs by Knocking Out Key Genes in the MicroRNA Biogenesis Pathway

MicroRNAs play an important role in many different cell types by negatively regulating gene expression. To explore the effect of microRNAs in Treg development and function, several groups established different gene knock-out mouse models. In 2006, a mouse model with a *Dicer* depletion in the CD4^+^ T lymphocytes was developed and exhibited immune injury of multiorgan including the colon, the lungs, and the liver. Absence of Dicer impairs nTreg in the thymus, resulting in fewer nTregs and reduced induction of Foxp3 in the CD4^+^CD25^−^ T cells in the periphery [30]. Subsequently, using a Foxp3-cre-mediated Dicer deletion mouse model, Liston et al. demonstrated that the Dicer-deficient Tregs isolated from healthy mice had an impaired proliferation capacity, increased apoptosis, and impaired suppressive function. However, under inflammatory conditions, the Dicer-deficient Tregs increased in number, but almost lost their suppressive capacity [31]. This indicated that microRNAs played an indispensable regulatory role in Tregs under both healthy and diseased conditions. In another Dicer knock-out model generated from a Treg-specific FoxP3-GFP-hCre bacterial artificial chromosome transgenic mouse, Tregs developed normally in the thymus, but exhibited an impaired differentiation and dysfunction in the periphery. These mice developed a systemic autoimmune disease, which was similar to the phenotype of Foxp3 knock-out mice [32]. This demonstrated that microRNA depletion impaired the Treg function and disrupted the immune homeostasis. Chong et al. created a mouse model with another miRNA-generating RNase III enzyme, Drosha, depleted in CD4^+^ T cells. This work demonstrated the number of Tregs and their Foxp3 expression were both reduced, and their suppressive capacity was weakened [33]. Dicer depletion not only disrupts cranial microRNA biogenesis but also the biogenesis of other small RNAs. DGCR8, which is an RNA-binding protein and necessary for microRNA processing, is more specific for microRNA generation than that observed for Dicer. To explore the role of microRNAs in Treg, a mouse model was generated with DGCR8 ablated specifically in Tregs. This model developed a spontaneous scurfy-like lethal syndrome and had reduced expression of Foxp3 in DGCR8-deficient Tregs *in vivo* and *in vitro* [34]. Together, this research demonstrates an indispensable regulatory role for microRNAs in Tregs, opening the possibility of the further research into the microRNA signature and the role of individual microRNAs in Tregs.

## 5. MicroRNA Profile of Tregs

The microRNA profile of Tregs could help distinguish them from other cells and provide cues for further research. Until now, only several studies have reported microRNA profiles of Tregs in human and mouse. In 2006, Cobb et al. first investigated differentially expressed microRNAs between nTregs and the conventional CD4^+^CD25^−^ T cells in mice by microarray. Their results identified 35 microRNAs that were upregulated in Tregs, including miR-223, miR-146, miR-21, miR-22, miR-23a and b, miR-24, miR-214, and miR-155 and 33 microRNAs that were downregulated, including miR-142-5p and miR-142-3p, miR-30b, miR-30c, miR-30e, and members of the Let-7 family [30]. In 2009, Rouas et al. first described the human nTreg microRNA signature, which was composed of five differentially expressed microRNAs (21, 31, 125a, 181c, and 374) [35]. Sadlon et al. investigated microRNAs regulated by Foxp3 that were differentially expressed between nTregs and Th cells isolated from the human cord blood. Seven microRNAs were preferentially expressed in nTregs, including mir146a, mir155, hsa-let7, mir101, mir7, mir21, and mir142-5p, while two microRNAs were downregulated in: mir19b and mir20b [36]. Subsequently, a microRNA profile of the circulating CD4-positive regulatory T cells in human adults was revealed. Compared with CD4^+^CD25^−^ T cells, mir95 and mir509 were upregulated in the CD4^+^CD25^+^CD127^low^ Treg, while the following thirteen microRNAs were downregulated: miR-9, miR-18a, miR-24, miR-27b, miR-126, miR-133a, miR-134, miR-145, miR-181b, miR-181d, miR-210, miR-224, and miR-335 [37]. However, these results are primarily based on microarray analyses and require experimental validation and functional analysis of individual microRNAs.

## 6. Roles of Individual MicroRNAs in Proliferation, Differentiation, and Function of Tregs

**Figure 2:**
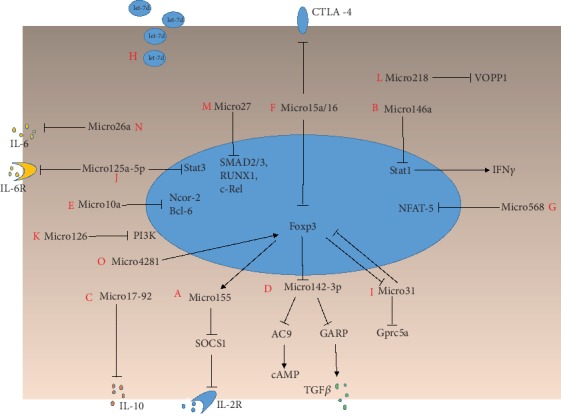
The regulatory roles of microRNAs in Tregs. A—Foxp3 directly regulates the expression of micro155. Micro155 directly targets the 3′ UTR of the mRNA of suppressor of cytokine signaling 1 (SOCS1), which suppresses IL-2 signaling. B—Micro146a negatively regulates IFN*γ* production by directly targeting its key transcription factor Stat1. C—Micro17-92 regulates secretion of the immunosuppressive cytokine IL-10. D—The expression of micro142-3p is negatively regulated by Foxp3 directly or indirectly. Micro142-3p inhibits the production and secretion of cAMP and TGF*β* by directly targeting adenylyl cyclase (AC) 9 and glycoprotein A repetitions predominant (GARP), respectively. E—Micro10a regulates Treg stability by targeting the transcriptional repressor Bcl-6 and the corepressor Ncor2. F—Micro15a/16 negatively regulates the expression of Foxp3 and CTLA-4. G—Micro568 regulates the activation, differentiation, and function of Tregs by targeting NFAT5. H—Let-7d plays an important role in the exosome-mediated immunosuppressive function of Tregs. I—Micro31 regulates periphery Treg generation by targeting Gpra5a. J—Micro125a-5p regulates the differentiation and function of Tregs by targeting IL-6R and Stat3. K—Micro126 influences the induction and suppressive function of Tregs through the PI3K/AKT pathway. L—Micro218 regulates the amount of Tregs by targeting VOPP1. M—Micro27 impairs the development and homeostasis of Tregs by targeting SMAD2/3, RUNX1, and c-Rel. N—Micro26a plays a vital role in the expansion and function of Tregs by targeting IL-6. O—Micro4281 promotes Tregs generation by binding to the TATA box of promoter of Foxp3.

Elucidating the roles of specific microRNAs in Tregs is necessary to understand the concrete regulatory mechanisms of Tregs by microRNAs and may provide another perspective to research immune-related diseases (Figure 2).

### 6.1. Micro155

Micro155 is highly expressed in Tregs and its expression is regulated directly by the Foxp3 transcription factor through binding to the promoter region of its host gene *bic* [38]. In mice depleted of micro155, the number of Tregs was reduced in both the thymus and spleen, but their suppressive capacity was not evidently altered [38]. Further work has revealed that micro155 is required for Treg development and is indispensable for proliferation or survival of Tregs in the thymus and periphery [38]. However, no micro155-deficient mice developed a spontaneous inflammatory bowel disease (IBD) [38]. Lu et al. confirmed that the proliferative capacity of micro155-deficient Tregs was reduced and Treg homeostasis was impaired in the absence of micro155 [39]. They discovered that micro155 conferred Tregs with an advantage in competition for the limited growth factor IL-2 which has been proven to be necessary for Treg proliferation [40]. Suppressor of cytokine signaling 1 (SOCS1), which suppresses IL-2 signaling, is a direct target of micro155 as shown by luciferase reporter experiments [39]. In SOCS1 transgenic mice, the number of Tregs was reduced, which was analogous to that in the miR155-deficient mice [39]. In conclusion, micro155 could affect IL-2 signaling by inhibiting SOCS1 expression to regulate Treg proliferation but has little effect on the suppressive function of Tregs.

### 6.2. Micro146a

Among the microRNAs differentially expressed between nTregs and conventional CD4^+^CD25^−^T cells, micro146 was evidently upregulated in Tregs [30]. Micro146a, which is potentially regulated by Foxp3, was found to be upregulated in human nTregs [36]. Lu et al. also confirmed that micro146a expression in the mouse CD4^+^CD25^+^Tregs is much higher than that in the CD4^+^CD25^−^CD62L^hi^-naive T cells [41]. To explore the role of micro146a in Tregs, micro146a-deficient mice were developed and exhibited serious lymphoproliferative and myeloproliferative syndrome at 6 months of age [41]. In the micro146a-deficient mice, the number of Foxp3^+^ Tregs was reduced in the periphery, but not in the thymus [41]. Using micro146a^−/−^/Foxp3KO chimeras, researchers demonstrated that a lack of micro146a could increase Treg numbers in a cell-autonomous manner, but led to an impaired Treg function [41]. *In vitro*, experiments indicated that micro146a deficiency in Tregs did not change their overall suppressive function. Increased proinflammatory Th1 cytokine IFN*γ* was observed in micro146a-deficient Tregs. Further analysis showed that Stat1, which is a key transcription factor in the IFN*γ* receptor signaling pathway, is a direct target of micro146a as shown by luciferase reporter experiments. Stat1 expression was increased in micro146a-deficient Tregs [41]. In conclusion, micro146a is indispensable for the Treg-mediated suppression of the IFN*γ*-dependent Th1 response by a negative regulation of the Stat1 expression.

### 6.3. Micro17-92

The micro17-92 polycistronic miRNA gene is transcribed as a primary transcript with the potential to generate six mature microRNAs including miR-17, miR-18a, miR-19a, miR-20a, miR-19b, and miR-92. Micro17-92 is reported to inhibit the TGF-induced Treg generation *in vitro* [42]. To investigate the role of micro17-92 *in vivo*, mice were generated with micro17-92 depleted specifically in Tregs. However, there were no signs of disease in the mice, indicating that micro17-92 is dispensable for Tregs under healthy conditions [42]. To further analyse the role of micro17-92 in Tregs under disease stress, the Treg^miR17-92-/-^ mouse model and controls were treated with myelin oligodendrocyte glycoprotein (MOG) emulsified in complete Freund's adjuvant (CFA) to induce EAE. The Treg^miR17-92-/-^ mice developed much more serious phenotypes than those in the controls. The proliferative activity of miR-17-92 deficient Tregs was not weakened *in vivo*. The overall numbers of Tregs in Treg^miR17-92-/-^ mice under disease stress were not evidently altered but the number of antigen-specific Tregs was reduced, which may be due to increased cell death. There was also a reduced proportion of IL-10 secreting Tregs, suggesting that micro17-92 may regulate immunosuppressive IL-10 secretion in Tregs [42]. Therefore, micro17-92 is not essential for Treg regulation under noninflammatory status but plays an important role in EAE disease by preserving antigen-specific Tregs and regulating IL-10 secretion in Tregs.

### 6.4. Micro142-3p

Tregs contain high levels of a ubiquitous secondary messenger, cyclic AMP (cAMP), and can exert their immunosuppressive role by transferring cAMP to recipient T lymphocytes through the gap junction channels [43]. cAMP can be produced by adenylyl cyclase (AC). Research by Huang et al. revealed that micro142-3p, which is downregulated in Tregs, could negatively regulate cAMP production by targeting AC 9 [44]. Overexpression of micro142-3p could impair the inhibitory effect of Tregs. Further research indicated that micro142-3p was negatively regulated by Foxp3 directly or indirectly [44]. These results stated that micro142-3p could affect Treg function by negatively regulating the AC9/cAMP pathway. Zhou et al. confirmed that glycoprotein A repetitions predominant (GARP) was another target of micro142-3p [45]. GARP is specifically expressed in activated Tregs and can modulate cell proliferation [45, 46]. Tregs transfected with mimics of micro142-3p showed a reduction of proliferation, in line with an increase of micro142-3p and a diminution of GARP, while there was a significant increase in proliferation in Tregs treated with an antagomir of micro142-3p [45]. GARP can also mediate suppressive signals, induce Foxp3 expression, and facilitate secretion of the inhibitory cytokine transforming growth factor-*β* (TGF-*β*), which indicates that micro142-3p may regulate Treg function by downregulating GARP through other mechanisms [46, 47]. In conclusion, micro142-3p modulates the proliferation and function of Tregs by targeting AC9 and GARP through distinct mechanisms.

### 6.5. Micro10a

Micro10a is highly expressed in nTregs and its expression can be induced by retinoic acid (RA) and TGF-*β* in iTregs [48]. The conversion of iTregs into follicular helper T cells is diminished by micro10a via targeting of the transcriptional repressor Bcl-6 and the corepressor Ncor2, showing that micro10a is required to maintain Treg stability [48]. Jeker et al. reported that micro10a is specifically expressed in Tregs and that micro10a inhibition can result in reduced Foxp3 expression *in vitro* [49]. However, in mice depleted of micro10a, neither the number or function of Tregs nor the ability of conventional T cells to be induced into iTregs by RA and TGF-*β* was evidently changed [49].

### 6.6. Micro15a/16

Micro15a/16 is expressed at much lower levels in the umbilical cord blood- (CB-) derived Tregs than those in the conventional CB T cells (Tcons) [50]. Overexpression of micro15a/16 in CB Tregs reduced expression of Foxp3 and CTLA4 and diminished the immunosuppressive function of Tregs [50]. Inhibition of micro15a/16 in CB Tcons not only led to Foxp3 and CTLA4 expression but also conferred immunosuppressive capacity to Tcons [50]. Therefore, micro15a/16 is required for Treg function and may play a “toggle-switch”-like role between Treg and Tcon, which needs further investigation *in vivo* [50]. Micro15a/16 and micro15b/16 both belong to the micro15 cluster and target the same mRNA sequence. Micro15b/16 is more abundant in Tregs than other types of T cells in mice [51]. Overexpression and blocking experiments showed that micro15b/16 could induce Tregs *in vitro* and *vivo* [51]. Micro15b/16 influence Treg induction through directly targeting Rictor, the mTORC2 component, and mTOR [51]. The difference of expression and role of micro15a/16 and micro15b/16 may be due to species variation in these studies and the experimental systems.

### 6.7. Micro568

Li et al. discovered that micro568 expression was reduced during Treg activation [52]. Transfection of Tregs with micro568 mimicked inhibited Treg activation, reduced TGF-*β* and IL-10 production, and diminished their proliferative capacity [52]. Further research found that micro568 overexpression in Tregs could limit differentiation and immunosuppressive function of Tregs [52]. Nuclear factor of activated T cells 5 (NFAT5) is a direct target of micro568 and transfection with siRNA-NFAT5 inhibits the activation and differentiation of Tregs [52]. These results indicate that micro568 inhibits activation, differentiation, and function of Tregs by targeting NFAT5.

### 6.8. Microlet-7d

Okoye et al. reported that Foxp3^+^ Tregs could exert their inhibitory role on Th1 cells by secreting exosomes containing microRNAs [53]. Rab27a and Rab27b are both required for exosome release [54]. Tregs isolated from Rab27^ashen/ashen^Rab27b^–/–^ double knockout mouse failed to secrete exosomes and lost their immunosuppressive ability [53]. The transfer of micro155, let-7b, and let-7d from Tregs to effector T cells was observed. Further research demonstrated that overexpression of let-7d in Th1 cells reduced Th1 cell proliferation and IFN-*γ* secretion. Tregs treated with a let-7d inhibitor which secrete let-7d depleted exosomes could result in an impaired immune suppression *in vivo* and *vitro*. Together, this work shows that let-7d plays an important role in the exosome-mediated immunosuppressive function of Tregs.

### 6.9. Micro31

Foxp3 was confirmed to be a target of micro31 by luciferase reporter experiments and micro31 overexpression in Tregs reduced Foxp3 expression [35]. Recently, Zhang et al. confirmed that T cell receptor (TCR) signaling could induce micro31 expression, while Foxp3 negatively regulated micro31 expression by binding to its promoter [55]. Further analysis demonstrated that conditional deletion of micro31 in CD4^+^ T cells induced pTreg (periphery Treg) generation and ameliorated the severity of experimental autoimmune encephalomyelitis (EAE) *in vivo*. Gpra5a was confirmed to be a direct target of micro31 [55]. Gprc5a, which is also known as retinoic acid-inducible protein 3, contains two transcription factors binding sites: retinoic acid receptors (RARs) and retinoid X receptors (RXRs) of RA [56]. Gprc5a expression was lower in EAE mice than that in controls, consistent with the higher micro31 expression in EAE mice than that in controls [55]. Under inflammatory conditions, Gprc5a^−/−^ mice have fewer pTregs and exhibit increased EAE severity [55]. These results suggest that micro31 may affect EAE severity by regulating pTreg generation through targeting Gprc5a and may serve as a therapeutic target of EAE disease.

### 6.10. Micro125a-5p

Micro125a-5p was downregulated in CD4^+^ T cells of autoimmune diseases, such as systemic lupus erythematosus and Crohn's disease. To research the role of micro125a in autoimmune disease, Pan et al. developed micro125a knock-out mice. They found that both the differentiation and function of micro125a deficient Tregs were restrained *in vitro* and *vivo*, while other CD4^+^ T cells (Th1, Th2, and Th17) were not significantly affected. Micro125a directly targets the effector T lineage factors Stat3, Il13, and Ifng to influence Treg differentiation and function [57]. Li et al. also confirmed that micro125a-5p could decrease the sensitivity of Treg differentiation to inflammatory-like T cells mediated by IL-6 by directly targeting IL-6R and Stat3. The regulatory loops of GATA3/micro125a-5p/IL-6R and Stat3/Foxp3 are involved in IL-6-mediated conversion of Tregs [58]. Other works indicated that micro125a-5p expression was higher in Tregs from draining lymph nodes (PLNs) from autoimmune type I diabetes (T1D) subjects than that in the healthy controls [59]. CCR2 expression CCR2 in Tregs is inversely correlated with micro125a-5p expression. CCR2 is a direct target of micro125a-5p as shown by luciferase reporter assays. The CCR2 ligand (CCL2) is expressed in beta cells. Micro125a-5p upregulation in Tregs of T1D patients may result in reduced CCR2 expression in Tregs and impede the migration of Tregs in the pancreas, which may further disrupt immune tolerance [59].

### 6.11. Micro126

Micro126 is expressed in Tregs and expression is higher when Tregs are activated. Silencing micro126a reduces Treg induction, decreases Foxp3 expression, and impairs the suppressive function of Tregs *in vitro.* Silencing micro126 also impairs the suppressive function of Tregs *in vivo* [60]. P85*β* is a direct target of micro126, and studies have reported that micro126 may regulate the PI3K/AKT pathway by targeting P85*β*. Micro126a alters Foxp3 expression through the PI3K/AKT pathway in Tregs [61, 62].

### 6.12. Micro181a

Tregs transfected with micro181a mimics show enhanced suppressive function through the PI3K/Akt pathway. Micro181a has no effect on Treg proliferation. Micro181a upregulates expression of interleukin-10 (IL-10) and TGF-*β*. The mRNA levels of IL-10 and TGF-*β*in peripheral blood mononuclear cells (PBMCs) of allergic rhinitis (AR) mice treated with micro181a mimics were higher than those of AR mice treated with micro181a inhibitors. These studies showed that micro181a regulated the suppressive function of Tregs *in vitro* and *vivo* [63]. Serr et al. showed that micro181a expression was increased in CD4^+^ T cells from children with onset of autoimmunity. Micro181a inhibits the induction of Tregs through PI3K-mediated NFAT5 expression. Systemic administration of micro181a antagomir or NFAT5 inhibitor ameliorates islet autoimmunity in murine models [64].

### 6.13. Micro218

Micro218 expression was lower in the PBMCs and Tregs of 53 sepsis patients when compared to 20 healthy controls. In advanced sepsis patients, Tregs micro218 expression was lower than that of early septic Tregs. Micro218 expression was much lower in PBMCs of dead sepsis patients than that of surviving patients. VOPP1 is one of the direct targets of micro218. Venous injection of micro218 mimics into a sepsis mouse model reduces the amount of Tregs, upregulates micro218 expression and, and downregulates VOPP1 expression [65]. This indicates that micro218 may regulate the amount of Treg cells and play an important role in the pathologic process of sepsis.

### 6.14. Micro27

Micro27 is a member of the miR-23~27~24 family and is upregulated in T cells from multiple sclerosis patients [66]. Further study demonstrated that mice with T cell-specific micro27 overexpression showed pathogenic Th1 responses and autoimmune pathogeny [67]. The abnormal immune reactions were driven by Treg dysregulation to some extent. Excessive micro27 expression in T cells could result in fewer Tregs. It was demonstrated that excessive micro27 impaired the development and homeostasis of Tregs by targeting SMAD2/3, RUNX1, and c-Rel, which are members of the NF-*κ*B transcription factor and crucial for Treg biology. At the same time, overexpressed micro27 could inhibit Treg function by targeting IL-10 and GZMB [67]. Another study demonstrated that micro27 was abundantly expressed in Tregs in mice, and overexpression weakened Treg generation [68]. In conclusion, micro27 plays an important role in the development, homeostasis, and function of Tregs.

### 6.15. Micro26a

Micro26a expression was lower in the peripheral blood lymphocytes of multiple sclerosis (MS) patients, especially in relapsing MS patients, than healthy controls [69]. In an EAE mouse model, expression of micro26a and Foxp3 was significantly lower in the acute phase, which was similar with relapsing MS patients. Overexpression of micro26a may upregulate Foxp3; in contrast, IL-6 overexpression could suppress micro26a-induced upregulation of Foxp3. Luciferase reporter assays showed that IL-6 is a direct target of micro26a. That is to say, micro26a may upregulate the function of Tregs by targeting IL-6, to some extent. Golshayan showed that micro26a could promote the expansion of Tregs in a mouse skin transplantation mouse model [70]. Together, this suggests that micro26a may play a vital role in the expansion and function of Tregs.

### 6.16. Micro466a-3p

Becker et al. analysed the microRNA expression profile of CD4^+^ T cells from the draining lymph node of a murine model of allogenic transplantation and found that micro466a-3p expression was increased after allogeneic transplantation [71]. To further research the role of micro466a-3p in Treg, micro466a-3p was transfected into CD4^+^ T cells, which could reduce iTregs, while inhibiting micro446-3p expression in CD4^+^ T cells could increase Tregs and reduce effector cells. Luciferase reporter assays showed that TGF-*β*2 was a direct target of micro466a-3p. Inhibition of micro466a-3p in an allogeneic skin-graft model abates the T cell response to the graft through the increased expression of TGF-*β*2. These results demonstrate the role of micro466a-3p in Treg differentiation.

### 6.17. Micro4281

Micro4281 is specifically expressed in hominids and specifically binds the TATA box in the Foxp3 promoter. The expression of micro4281 was much higher in Tregs than that of naïve T cells sorted from human PBMCs [72]. Experiments showed that micro4281 enhanced the promoter activity and transcription of Foxp3 and facilitated Treg development [72]. Overexpression of micro4281 significantly induces naïve T cells to Tregs. Micro4281 mimics ameliorate the graft versus host disease (GVHD) in a mouse model. IL2 signaling is essential for Treg development. IL2/STAT5 signaling induces micro4281 expression by binding to the promoter of SNCB, which then promotes Foxp3 expression [72]. In conclusion, micro4281 plays an important role in the development of Tregs by binding to the TATA box of the Foxp3 promoter.

### 6.18. Other MicroRNAs

Among the nTreg microRNA signature (micro21, micro31, micro125a, micro181c, and micro374), micro21 and micro31 were shown to regulate Foxp3 expression, while the other three microRNAs had no effect of Foxp3 expression and Treg phenotypes [35]. Compared with conventional T cells, micro21 was upregulated and micro31 was downregulated in Tregs. Micro21 positively regulates Foxp3 expression indirectly, and micro21 overexpression in non-Tregs can induce Foxp3 expression [35]. In the microRNA profile of circulating CD4^+^ Tregs in human adults, several microRNAs were confirmed to regulate Foxp3 and CTLA4 expressions [37]. Micro24 and micro210 were underexpressed in Tregs and could negatively regulate Foxp3 expression by directly binding to its 3′ UTR [37]. Overexpression of micro24 and micro210 in Tregs could lead to a reduced Foxp3 expression. However, other research demonstrated that micro24 expression was high in mouse Tregs, and blocking and overexpression experiments showed micro24 could induce Treg generation and differentiation by directly targeting IL-4 [51, 68]. The different influences of micro24 on Tregs shown in the abovementioned studies may be due to experimental differences in each study. Micro95 is upregulated in Tregs, positively regulates Foxp3 expression indirectly, and micro95 overexpression in CD4^+^CD25^−^ T cells increases Foxp3 expression [37]. Micro145 is downregulated in Tregs, inhibits CTLA4 directly by binding to its 3′ UTR, and micro145 overexpression in Tregs reduces CTLA4 expression [37]. Micro23 and micro29a are both abundantly expressed in mouse Tregs. Micro23 overexpression inhibits Treg generation; in contrast, micro29a overexpression induces Tregs generation. Kaul et al. transfected peripheral blood mononuclear cells (PBMCs) with micro2909, which led to an evident increase of CD4^+^CD25^+^Foxp3^+^ Tregs among the CD4^+^ T population [73]. This indicates that micro2909 may play a regulatory role in Treg proliferation, but this requires further investigation. Together, these studies demonstrate that micro21, micro23, micro31, micro24, micro29a, micro95, micro145, micro210, and micro2909 are microRNAs with the potential to regulate the development, proliferation, and function of Tregs and further research may confirm their roles in immune regulation.

## 7. The Regulatory Role of MicroRNAs in Tregs: Learning from Human Diseases and Animal Disease Models

Due to the regulatory role of microRNAs in Tregs, altered microRNA expression in Tregs may play an important role in the occurrence and development of diseases by disrupting immune homeostasis. Here, we summarized microRNAs with altered expression in Tregs in human diseases and animal disease models (Table 1).

### 7.1. Micro210

Zhao et al. reported that micro210 expression was evidently increased in CD4^+^ T cells isolated from patients with Psoriasis vulgaris (PV), a chronic inflammatory and autoimmune skin disease [74]. Foxp3 was confirmed to be a micro210 target by luciferase reporter experiments, which was consistent with previous studies [37, 74]. CD4^+^ T cells from PV patients have impaired immune function [74]. Transfection of CD4^+^ T cells from healthy controls with micro210 reduced Foxp3 expression and impaired the immunosuppressive function of Tregs [74], whereas micro210 inhibitor-treated CD4^+^ T cells from PV patients increased Foxp3 expression and improved immune dysfunction [74]. This work indicates that an increased micro210 in PV patients downregulates Foxp3 expression in Tregs, affecting Treg function and immune homeostasis.

### 7.2. Micro10a and Micro182

In *Schistosoma mansoni*-infected mice, which evoke Th2 responses, micro182 expression in Foxp3^+^ Tregs was much higher than that in controls [75]. Micro182 is required for Treg-mediated Th2 proliferation *in vitro* and Th2-driven airway inflammation *in vivo* by controlling IL-2 production of Tregs [75]. Leishmania-infected mice, which evoke Th1-mediated responses, showed significant underexpression of micro10a in Tregs compared with controls [75]. Micro10a is essential for Treg-mediated Th1 proliferation *in vitro* and Th1-driven inflammation *in vivo* by regulating IFN*γ* production [75].

### 7.3. Micro214

Micro214 is an important oncogenic microRNA that promotes tumor growth by targeting and downregulating phosphatase and tensin homolog (PTEN) in human ovarian cancer [76]. PTEN is a confirmed negative regulator of Treg expansion. Recent research has demonstrated that cancer-secreted micro214 could be transferred into recipient T cells by microvesicles [77]. In mouse tumor models, cancer-secreted micro214 significantly inhibits PTEN expression and promotes Treg expansion. Micro214-induced Tregs lead to immune suppression and tumor growth. In contrast, anti-micro214 antisense oligonucleotides are able to block Treg expansion and tumor growth in a mouse tumor model [77]. These results demonstrate that micro214 inhibits Treg expansion by targeting PTEN.

### 7.4. Micro155, Micro21, and Micro31 in Kawasaki Disease

Kawasaki disease (KD) is an acute systemic vasculitis syndrome and is likely to occur in infants and children. Previous studies have shown immune dysfunction is involved in KD pathogenesis and that the numbers and function of Tregs are reduced in KD [78–80]. Recent studies have indicated that micro155 and micro21 expressions were downregulated and micro31 expression was higher in KD patients. Previous studies demonstrated that micro155 and micro21 positively regulated Foxp3 expression, and micro31 inhibited Foxp3 expression by directly targeting Foxp3 mRNA [35, 39]. Multiple linear regression analysis of microRNA and Foxp3 mRNA levels of KD patients indicated that micro155 and micro31 control FoxP3 mRNA levels [81]. This suggests that micro155 and micro31 may play important roles in KD development.

### 7.5. Altered MicroRNA Expression Profile of Tregs in Multiple Sclerosis

Multiple sclerosis (MS) is a type of autoimmune disease. Previous studies have demonstrated that CD4^+^CD25^+high^ Tregs from MS patients lose their immunosuppressive function, which contributes to immune imbalance [82–84]. De Santis et al. assessed microRNA expression profiles of Tregs from MS patients and controls. They found that the expression of micro25, micro19a, micro106b, micro19b, micro93, and micro210 in Tregs from MS patients was much higher than that of controls [85]. Among them, micro25 and micro106b silenced the cell cycle inhibitor CDKN1A (p21) and the proapoptotic gene *BCL2L11* (BIM), which are important effectors in the TGF-*β* signaling pathway [86]. TGF-*β* is an important cytokine which plays important roles in Treg production and function [87, 88]. Therefore, micro25 and micro106b may regulate Treg function by interfering with the TGF-*β* signaling pathway and play important roles in the MS development. Micro210, which has been confirmed to target and inhibit Foxp3, was highly enriched in Tregs from MS patients [74]. This indicates that micro210 may exert its role on Treg function and MS pathogenesis by targeting Foxp3. Micro223 was significantly upregulated in CD4^+^ T cells from relapsing phase MS patients as compared to remitting and healthy individuals. At the same time, the cell marker of Th17 cells, ROR*γ*t, was upregulated in relapsing phase MS patients, whereas the expression of the Treg cell marker, Foxp3, was upregulated in remitting phase patients [89]. Micro223 may play a role in MS progression through regulating the balance of Th17 and Foxp3 expressions. Kimura et al. demonstrated that let-7i levels in exosomes of MS patients were much higher than that of healthy controls [90]. Let-7i could suppress the Treg generation by targeting insulin-like growth factor 1 receptor (IGF1R) and transforming growth factor beta receptor 1 (TGFBR1) [90]. This suggests that let-7i may play an important role in the development of MS by regulating the Treg induction.

### 7.6. Micro326

Micro326 has been shown to be upregulated in the peripheral blood mononuclear cells (PBMC) of ischemic stroke (IS) patients [91]. In another study, micro326 expression was significantly higher in the splenic CD4^+^ T cells than in the healthy controls [57]. This indicates that micro326 may play a role in autoimmune diseases. Sun et al. demonstrated that micro326 was upregulated in Tregs of systemic lupus erythematosus (SLE) patients [92]. Micro326 expression was negatively correlated with Ets-1 expression in Tregs. A previous study showed that Ets-1 deficiency could lead to Foxp3 upregulation in Tregs [93]. This indicates that micro326 may play a role in regulating Treg by targeting Ets-1; however, this needs further experimental evidence.

### 7.7. Micro342, Micro191, and Micro510 in Type 1 Diabetes

Tregs are an important regulator in type 1 diabetes (T1D). Becker et al. compared the differentially expressed microRNAs in Tregs of type 1 diabetes patients with healthy controls [94]. Micro510 expression in T1D patients was higher than that in healthy controls, whereas the expression of micro342 and micro191 was lower than controls [94]. The regulators of NF-*κ*b activation in diabetes, EP300, BMPR2, and PDGFRA, which are molecules involved in cytokine signaling, were predicted targets of micro342. The downregulation of micro342 in Tregs of T1D patients may influence the function of Tregs through these targets and should be verified by further study.

### 7.8. Micro31, Micro219, and Micro490 in Type 1 Delayed-Type Hypersensitivity

The delayed-type hypersensitivity (DTH) mouse model was made using methylated bovine serum albumin (mBSA). The expression of micro31, micro219, and micro490 was much higher in Tregs from draining lymph nodes (DLN) of DTH mice than that in controls [95]. Treatment of DTH with the aryl hydrocarbon receptor (AhR) ligands (indole-3-carbinol [I3C] and 3,39-diindolylmethane [DIM]) attenuates DTH response and promotes Treg generation. The expression of micro31, 219, and 490 was much lower in Tregs from DTH mice with treated I3C or DIM than that of untreated DTH mice. Analysis showed that micro31, micro219, and micro490 target the 3′ UTR of Foxp3. Further overexpression and blocking experiments showed that micro490 could decrease the expression of Foxp3 [95].

## 8. Conclusion

Tregs play important roles in immune balance and some immune-related diseases. MicroRNAs are small, noncoding RNAs and exert their regulatory roles in many cell types by targeting the 3′ UTR of coding genes. In recent years, many studies have revealed that microRNAs play an important regulatory role in Tregs. MicroRNAs not only regulate the function of normal Tregs but altered expression microRNA in Tregs could also lead to immune-related diseases. Therefore, inhibitors which target the microRNAs expressed abnormally in Tregs of immune diseases may be helpful to treat immune-related diseases. Though the roles of some microRNAs in Tregs are not well understood, they provide a basis for future research, which need *in vitro* and *in vivo* studies. With the current understanding of the roles of microRNAs in Tregs, we believe that microRNAs in Tregs may serve as therapeutic targets for immune-related diseases.

## Figures and Tables

**Table 1:** Altered expression of microRNA in Tregs in immune-related diseases.

MicroRNA	Human diseases or animal models	Expression in Treg	Influence on Treg	Target	Reference
Micro210	Psoriasis vulgaris	↑	Impair immune function	Foxp3	[56]
	Multiple sclerosis	↑	Lose immunosuppressive function	Foxp3	[56]; [80–83]
Micro10a	Leishmania-infected mice	↓	Treg-mediated Th1 proliferation	IFN*γ*	[73]
Micro182	Schistosoma mansoni-infected mice	↑	Treg-mediated Th2 proliferation	IL-2	[73]
Micro214	Ovarian cancer	↑	Promote Treg expansion	Phosphatase and tensin homolog (PTEN)	[74]
Micro155	Kawasaki disease	↓	Reduce generation and function	Positively regulating Foxp3 expression	[76–79]
Micro31	Kawasaki disease	↑	Reduce generation and function	Foxp3	[76–79]
Micro25	Multiple sclerosis	↑	Lose immunosuppressive function	TGF signaling pathway	[83–86]
Micro106b	Multiple sclerosis	↑	Lose immunosuppressive function	TGF signaling pathway	[69–73]
Let-7i	Multiple sclerosis	↑	Suppress Treg generation	IGF1R and TGFBR1	[90]
Micro490	Delayed-type hypersensitivity (DTH) mouse model	↑	Suppress Treg generation	Foxp3	[95]
